# Chemopreventive Efficacy of Atorvastatin against Nitrosamine-Induced Rat Bladder Cancer: Antioxidant, Anti-Proliferative and Anti-Inflammatory Properties

**DOI:** 10.3390/ijms13078482

**Published:** 2012-07-09

**Authors:** Belmiro Parada, Flávio Reis, Ângela Pinto, José Sereno, Maria Xavier-Cunha, Paula Neto, Petronila Rocha-Pereira, Alfredo Mota, Arnaldo Figueiredo, Frederico Teixeira

**Affiliations:** 1 Laboratory of Pharmacology & Experimental Therapeutics, Institute of Biomedical Research on Light and Image, Medicine Faculty, Coimbra University, Coimbra, 3000-548, Portugal; E-Mails: lipa.bq@gmail.com (Â.P.); jose6sereno@hotmail.com (J.S.); 2 Department of Urology & Renal Transplantation, Coimbra University Hospital, Coimbra, 3000-075, Portugal; E-Mails: afmota@iol.pt (A.M.); ajcfigueiredo@gmail.com (A.F.); 3 Service of Anatomic Pathology, Coimbra University Hospital, Coimbra, 3000-075, Portugal; E-Mails: mfxaviercunha@huc.min-saude.pt (M.X.-C.); anahepafr@sapo.pt (P.N.); 4 Research Centre for Health Sciences, Beira Interior University, Covilhã, 6201-506, Portugal; E-Mail: petrorp@ubi.pt

**Keywords:** bladder cancer, chemoprevention, atorvastatin, antioxidant, anti-proliferative, anti-inflammatory

## Abstract

To investigate the anti-carcinogenic effects of Atorvastatin (Atorva) on a rat bladder carcinogenesis model with *N*-butyl-*N-*(4-hydroxibutil)nitrosamine (BBN), four male Wistar rat groups were studied: (1) Control: vehicle; (2) Atorva: 3 mg/kg bw/day; (3) Carcinogen: BBN (0.05%); (4) Preventive Atorva: 3 mg/kg bw/day Atorva + BBN. A two phase protocol was used, in which the drug and the carcinogen were given between week 1 and 8 and tumor development or chemoprevention were expressed between week 9 and 20, when the bladders were collected for macroscopic, histological and immunohistochemical (p53, ki67, CD31) evaluation. Serum was assessed for markers of inflammation, proliferation and redox status. The incidence of bladder carcinoma was: control 0/8 (0%); Atorva 0/8 (0%); BBN 13/20 (65%) and Atorva + BBN 1/8 (12.5%). The number and volume of tumors were significantly lower in the Atorva + BBN group, with a marked reduction in hyperplasia, dysplasia and carcinoma *in situ* lesions. An anti-proliferative, anti-inflammatory and antioxidant profile was also observed in the preventive Atorva group. p53 and ki67 immunostaining were significantly increased in the BBN-treated rats, which was prevented in the Atorva + BBN group. No differences were found for CD31 expression. In conclusion, Atorvastatin had a clear inhibitory effect on bladder cancer development, probably due to its antioxidant, anti-proliferative and anti-inflammatory properties.

## 1. Introduction

In developed nations, bladder cancer is the fourth most common tumor in men and the eighth in women, accounting for 5–10% of all malignancies in men [1]. It is associated with high incidence and prevalence rates, and elevated socioeconomic costs [2,3]. Furthermore, it presents high recurrence and progression rates are a poor prognosis, particularly when late diagnosed or inadequately treated [1,3,4]. Despite current efforts on earlier diagnosis and more aggressive treatments, mortality rates in muscle-invasive disease are still very high [5]. In this context, preventive strategies will be pivotal for the management and treatment of bladder cancer, which depends on a better elucidation of the molecular mechanisms underlying appearance and growth.

Bladder tumor is associated with exogenous risk factors, which include mainly the cigarette smoking habit, present in more than 50% of the cases in the male population [3,6], and the exposure to occupational carcinogens, in particular aromatic amines and polycyclic aromatic hydrocarbons [6,7]. However, besides these risk factors and some genetic features already characterized [8,9], the putative involvement of inflammatory, proliferative and oxidative stress pathways deserve proper clarification. The rat bladder cancer induced by *N*-butyl-*N*-(4-hydroxybutyl) nitrosamine (BBN) is an appropriate and validated model to study human cancer development [10]. Due to the histological similarities with the human bladder cancer, it has been the most used model for the study of tumor pathophysiology, as well as to evaluate the efficacy of therapeutic strategies [10–12]. The urothelial carcinogenesis is a continuous and slow process that that goes through molecular and morphological changes, from benign to aggressive lesions. Thus, an early treatment targeting these pathways could hypothetically prevent bladder cancer development and growth. Previous reports have demonstrated beneficial effects of preventive strategies [13], including our own studies using anti-inflammatory and anti-proliferative agents [14,15].

During the last years, 3-hydroxy-3-methylglutaryl-coenzyme A (HMGCoA) reductase inhibitors (statins) have been indicated as potential anticancer agents [16–18]. Beyond their anti-dyslipidaemic properties, statins exhibit many other biological activities, known as pleiotropic effects [19]. Statins have been shown anti-inflammatory, anti-proliferative, antioxidant and anti-apoptotic properties [20–22], which could have an important role in bladder tumor prevention [23], particularly when considering that these pathways are up-regulated in this type of cancer [14,15]. The chemopreventive efficacy of statins was already demonstrated in other types of tumors, including in the pancreatic and the prostate cancers, resulting in retardation of tumor growth and/or inhibition of the metastatic process [24,25]. However, its action in bladder cancer remains to be proved, deserving further research.

Considering the beneficial effects of statins on the reduction for cardiovascular risk due to the antidyslipidemic effect, a positive impact against bladder cancer would be a major advantage, translating into a significant medical and socioeconomic benefit in patients that share several risk factors. Hence, this work intended to evaluate the chemopreventive efficacy of atorvastatin in a rat model of nitrosamine-induced bladder cancer.

## 2. Results

### 2.1. Bladder Cancer Development and Growth

No relevant changes were detected between the groups throughout the study concerning body weight and beverage consumption (data not shown). The lungs, stomach, liver, kidneys and intestines were normal on gross inspection and on histological examination, confirming the specificity of this model for bladder cancer development.

#### 2.1.1. Macroscopic Evaluation

**Figure 1:**
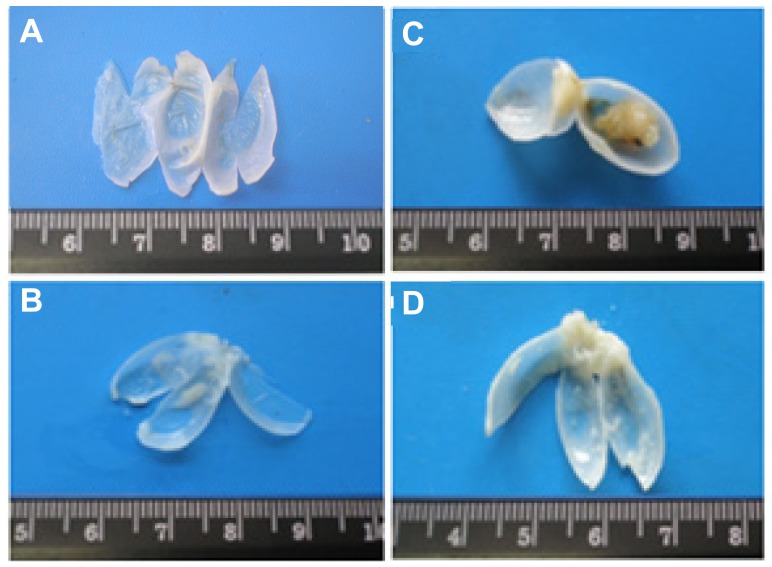
Macroscopic histomorphological evaluation of bladders, from the groups under study: (**A**) Control; (**B**) Atorva; (**C**) *N*-butyl-*N*-(4-hydroxybutyl)nitrosamine (BBN); (**D**) Atorva + BBN.

All bladders from the control animals were normal (Figure 1A). Similar profile was found for the Atorva group, with translucent and tiny bladders, without any abnormal mass or vascularization (Figure 1B). In the BBN group, however, 65.0% (13 in 20) of the rats had grossly visible bladder tumors, some of them of large dimensions (Figure 1C). The bladder walls were thicker than those of the control rats, with new or enlarged small vessels. In the Atorva + BBN group, all but one bladder showed a pattern similar to those of the controls (Figure 1D), the remaining bladder showing 2 small tumors.

#### 2.1.2. Quantitative Evaluation

In the control and Atorva groups, no tumors were seen. In the BBN group, 16 tumors were detected in 13 rats (with a mean of 1.2 ± 0.1 tumors per rat with tumor). In these 13 rats, the mean tumor volume was 138.5 ± 7.5 mm^3^ and the mean tumor volume per tumor was 112.5 ± 6.4 mm^3^ (Table 1). In the Atorva + BBN group, 1 (12.5%) of the 8 rats had 2 tumors with a summed volume of 4.7 mm^3^ and a mean tumor volume per tumor of 2.3 ± 0.2 mm^3^ (Table 1).

#### 2.1.3. Qualitative Evaluation

**Figure 2:**
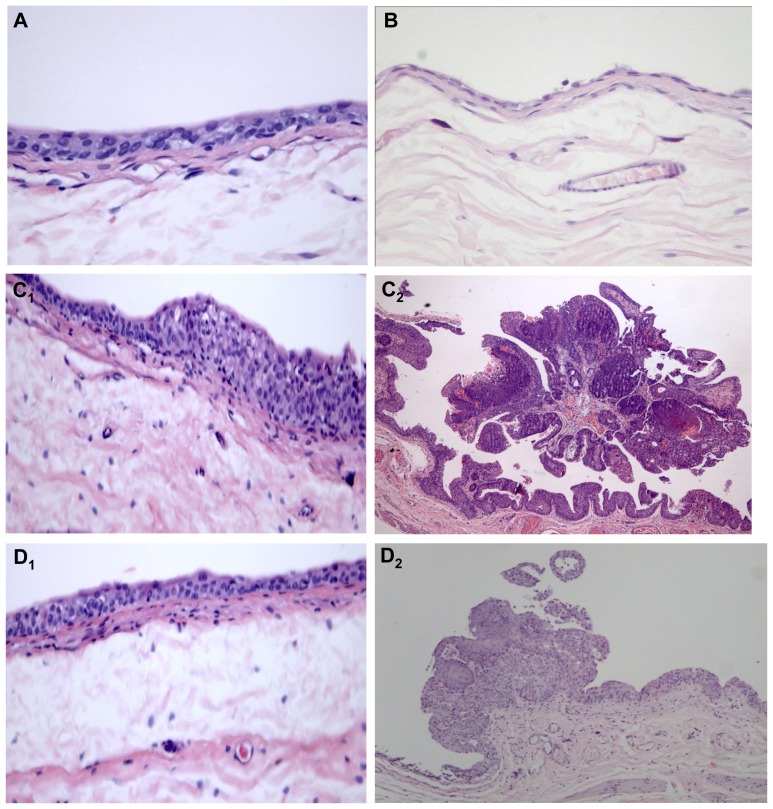
Microscopic histomorphology evaluation of bladders, from the groups under study: (**A**) Control; (**B**) Atorva; (**C_1_** and **C_2_**) BBN; (**D1** and **D2**) Atorva + BBN. H & E staining (100×).

Histological evaluation showed a normal urothelium in the control and in the Atorva rats (Figure 2A,B, respectively). No pre-neoplastic lesions (hyperplasia or dysplasia) were found (Table 1). In the carcinogen (BBN) group, there was evident malignant transformation, including hyperplasia (100%) and dysplasia (100%), present in all the animals, including those without tumors (Table 1 and Figure 2C_1_). Furthermore, there were also malignant lesions: papillary, infiltrative and carcinoma *in situ* (CIS) (Table 1 and Figure 2C_2_). The use of Atorvastatin (Atorva + BBN group) prevented bladder cancer development. Only one bladder (12.5%) of the 8 rats from this group has presented cancer development: two papillary tumors (Table 1). Histology also showed hyperplasia and dysplasia but no CIS or infiltrative lesions were identified (Table 1 and Figure 2D_2_). None of the other rats from this group had pre-malignant lesions and hyperplasia was found in only one case (Table 1 and Figure 2D_1_).

### 2.2. Systemic Proliferation, Inflammation and Oxidative Stress Markers

**Figure 3:**
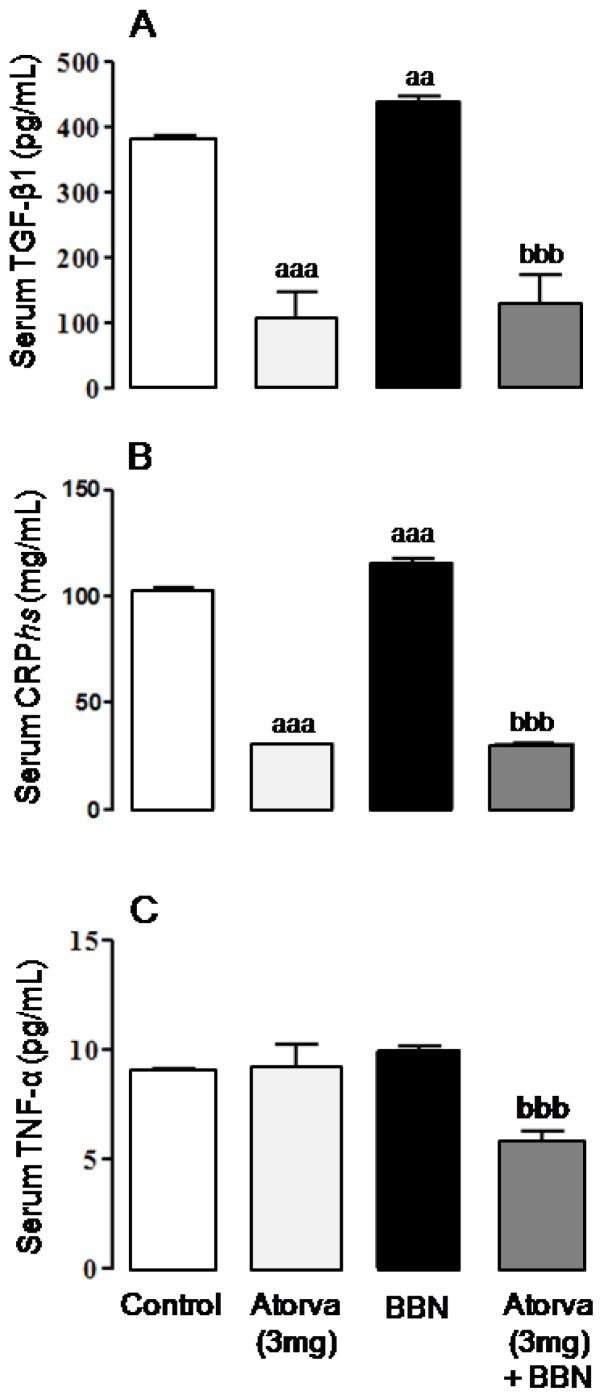
Serum markers of proliferation and inflammation. (**A**) TGF-β1; (**B**) CRP*hs*; (**C**) TNF-α. Values are mean ± SEM. a: *p* < 0.05 and aaa: *p* < 0.001 *vs.* Control group; bbb: *p* < 0.001 *vs.* BBN group.

In the BBN group there was a significant increment in serum transforming growth factor beta-1 (TGF-β1) (*p* < 0.01) and C-reactive protein (CRP) (*p* < 0.001), when compared with the control group (Figure 3A,B, respectively). Serum tumor necrosis factor alpha (TNF-α) levels were unchanged between BBN and control rats, despite a trend to higher values in the carcinogen group (Figure 3C). Compared to the control group, the Atorva-treated rats showed reduced concentrations of TGF-β1 and CRP (*p* < 0.001), whereas TNF-α was unchanged (Figure 3). In the Atorva + BBN group, the BBN-induced increment of serum levels of TGF-β1 and CRP was totally prevented (*p* < 0.001), together with a significant (*p* < 0.001) reduction of TNF-α contents (Figure 3).

**Figure 4:**
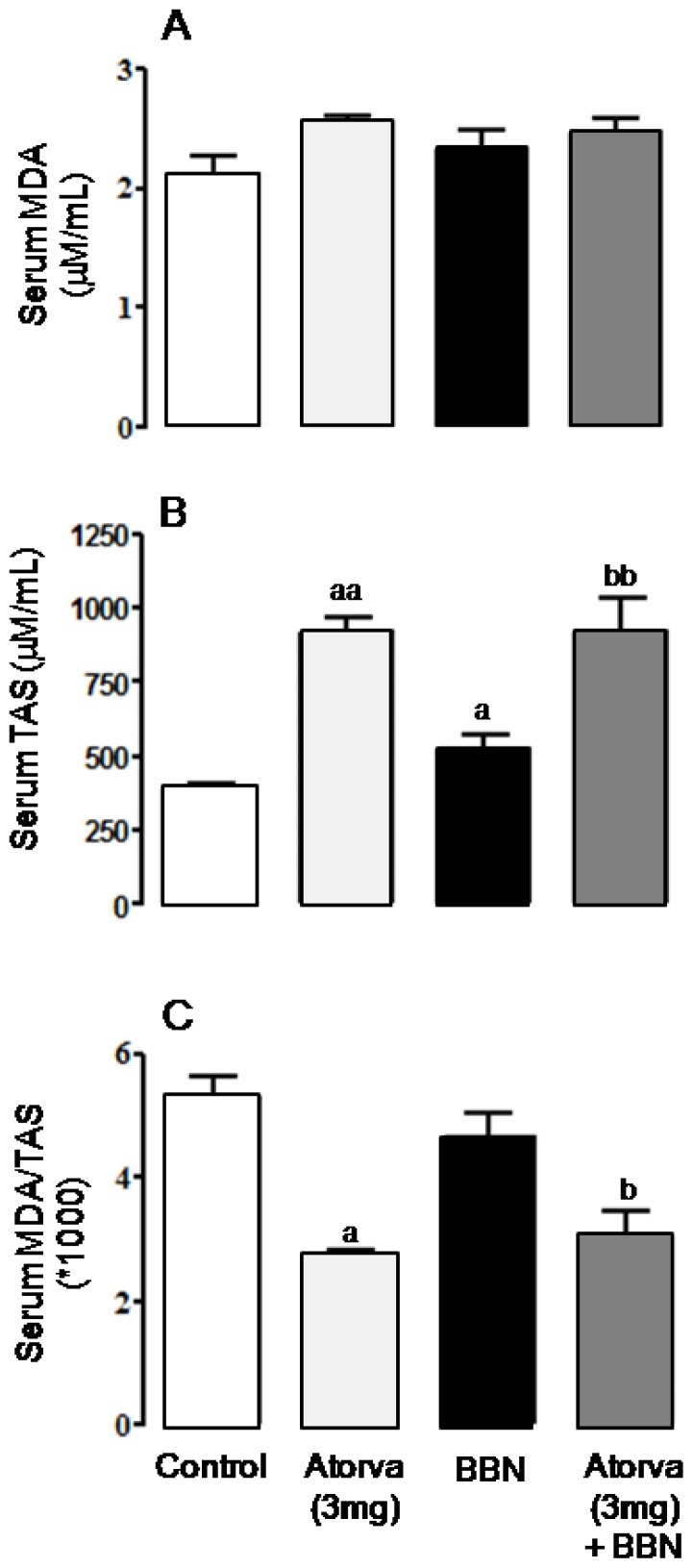
Serum redox status markers. (**A**) Lipidic peroxidation, via MDA content; (**B**) TAS levels; (**C**) MDA/TAS ratio. Values are mean ± SEM. a: *p* < 0.05, aa: *p* < 0.01 *vs.* Control group; b: *p* < 0.05, bb: *p* < 0.01 *vs*. BBN group.

Although non-statistically significant, serum malondialdehyde (MDA) concentration showed a trend to higher values in the BBN group when compared with the control, which was accompanied by a significant increase (*p* < 0.05) in serum total antioxidant status (TAS). In agreement, serum MDA/TAS ratio, a redox status marker, was unaltered (Figure 4). In the Atorva group, while serum MDA showed a trend to increased concentrations, there was a significant increase (*p* < 0.01) in serum TAS, accompanied by a significant reduction (*p* < 0.05) of serum MDA/TAS ratio, versus the control rats. Similar antioxidant profile was found for the Atorva + BBN group *vs.* BBN, in which there were significantly higher values of TAS (*p* < 0.01) and unchanged of MDA, resulting in a significantly lower ratio MDA/TAS (*p* < 0.05), demonstrative of an antioxidant profile (Figure 4).

### 2.3. Bladder Cancer p53, ki67 and CD31 Immunohistochemistry

**Figure 5:**
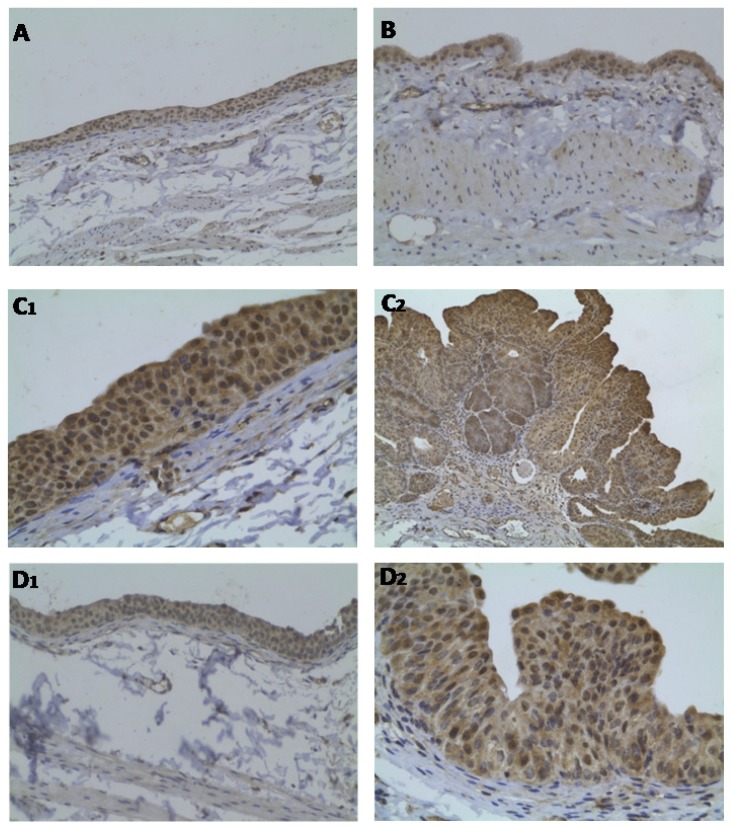
Bladder cancer p53 immunostaining, for the groups under study: (**A**) Control; (**B**) Atorva; (**C_1_** and **C_2_**) BBN; (**D_1_** and **D_2_**) Atorva + BBN. Original magnification 100× (**A**, **B** and **D_1_**) and 200× (**C_1_**, **C_2_** and **D_2_**).

The bladders from control (Figure 5A) and Atorva (Figure 5B) treated-rats presented normal p53 immunostaining intensity (grade 1). However, in the BBN group, both the hyperplastic (Figure 5C_1_) and tumoral (Figure 5C_2_) lesions showed elevated expression of p53 (grade 3 intensity). In the Atorva + BBN rats, all the bladders with normal urothelium (Figure 5D_1_) showed a p53 immunostaining identical to the control bladders (grade 1). The bladder with cancer (Figure 5D_2_) showed more intense p53 expression (grade 2) than those of the same group without tumor but lower intensity than those of the BBN rats.

**Figure 6:**
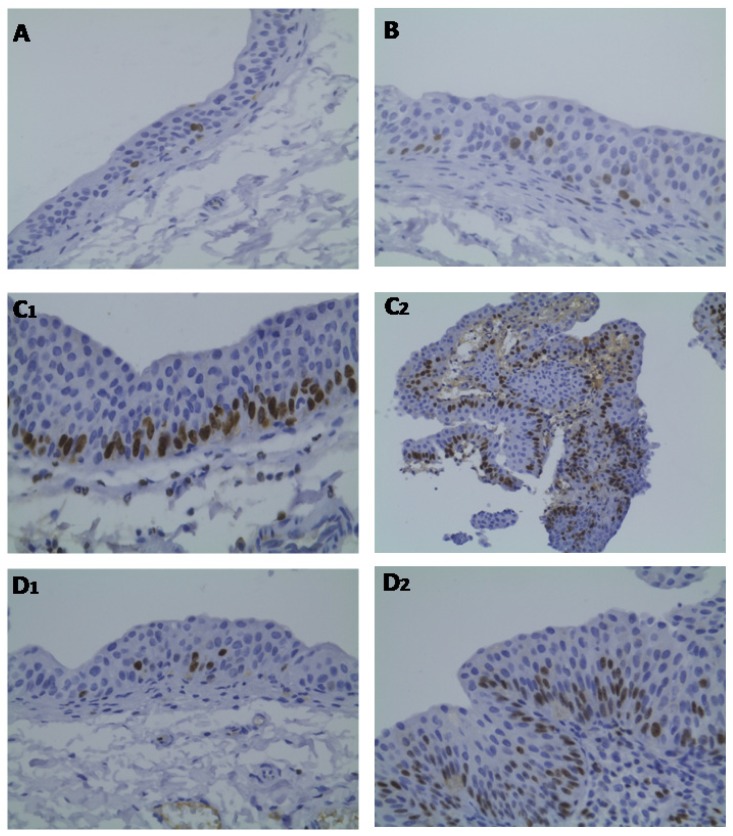
Bladder cancer ki67 immunostaining, for the groups under study: (**A**) Control; (**B**) Atorva; (**C_1_** and **C_2_**) BBN; (**D_1_** and **D_2_**) Atorva + BBN; Original magnification 100× (**A**, **C_2_** and **D_1_**) and 200× (**B**, **C_1_** and **D_2_**).

Regarding ki67 expression, reduced percentage of ki67 immunostaining was found in both the bladders from control (Figure 6A) and Atorva (Figure 6B) treated-rats (grade 0). In contrast, in the BBN group, the hyperplastic lesions (Figure 6C_1_) presented higher percentage of ki67 immunostaining (grade 1), which was even superior (grade 2) in the tumoral lesions (Figure 6C_2_). This pattern was prevented in the Atorva + BBN rats, with all bladders showing reduced ki67 expression: a low percentage (grade 0) was found in the bladders without tumor and a grade 1 in the bladder with tumours (Figure 6D_1_,D_2_, respectively). We did not find significant differences in CD31 (PECAM-1) expression among the groups under study (data not shown).

### 2.4. Biochemical Data: Safety Profile

In the BBN group, renal function markers (creatinine and urea) were unchanged; the liver function parameter AST was significantly (*p* < 0.001) increased; and there was a trend to higher values of creatine kinase (CK), when compared with the control group (Table 2). The Atorva group had a significant (*p* < 0.05) increase in CK activity versus the control group. In the Atorva + BBN group, the renal markers remained unchanged but there was an increment in AST and ALT (*p* < 0.001 and *p* < 0.01 *vs.* BBN, respectively), together with a higher CK value (*p* < 0.001 *vs.* BBN) (Table 2).

## 3. Discussion

During the last years, statins have been indicated as potential anticancer agents beyond their typical antihyperlipidaemic actions [16–18], particularly because of their pleiotropic effects [19]. Some medical-record database studies have shown significant statin-associated reductions in overall cancer incidence [16]. The chemoprevention properties of these drugs have been tested, with promising results, in several types of cancer [24–27]. However, investigation on its efficacy in bladder cancer prevention is almost non-existent [28]. Older and hyperlipidaemia patients are most effected by bladder cancer. Moreover, untreated hyperlipidaemia is associated not only with increased risk of bladder cancer development [29] but also with more aggressive forms [30]. Thus, if proved its anticancer properties, the preventive use of statins for those patients could have a double benefit, preventing both hypercholesterolaemia and cancer, namely bladder cancer.

The main finding of our study, not previously shown in any animal model (or in humans) is that tumor growth is inhibited by Atorva treatment. We found in the BBN + Atorva animals a tumour incidence of 12.5% (1 in 8 rats) with a mean tumor volume per tumor of 2.3 ± 0.2 mm^3^, contrasting with the data from the BBN rats: 65% incidence and 112.5 ± 6.4 mm^3^ mean tumor volume (in 16 tumors of 13 rats with tumor). Furthermore, apart from the rat with tumor, whose urothelium also showed hyperplasia and low-grade dysplasia, all other rats of the BBN + Atorva group had a normal urothelium (one of them showed low-grade hyperplasia). The hyperplasia and high-grade dysplasia induced by the carcinogen, present in all BBN-treated animals, were significantly reduced in the Atorva group, to 38% and 25%, respectively, together with an absolute prevention of infiltrative tumors and CIS. These data reinforces the notion that this drug is effective not only for chemoprevention of gross tumor appearance and growth but also for prevention of pre-neoplastic lesions.

This remarkable chemopreventive efficacy on tumor growth was achieved with what might be considered a low Atorva dose for animal studies (considering their pharmacokinetic differences with humans). This dosage is similar to the low-doses used in other recent studies with atorvastatin [31–33], reinforcing the promising results. Nevertheless, future research should confirm the drug blood concentrations achieved in order to assess their efficacy in bladder cancer in humans. Apart from the expected increment in CK activity, and some deterioration of hepatic (AST and ALT) markers, which may be due to the amplification of the carcinogen effects, Atorva treatment, particularly at low-doses, is unlikely to be associated with an unsafe profile. In any case, the elevations of CK and hepatic markers raise some concerns about the safety profile and deserve further study, including assessing if even lower doses of Atorvastatin in this animal model will be able to maintain the chemopreventive efficacy with a safe profile. Statins have a well known safety profile in the clinical setting, which is a clear advantage over other potential anti-carcinogen agents. Clinical trials have shown that statins are relatively well tolerated; therefore, gastrointestinal symptoms, abnormal liver enzyme levels and myalgia and/or myositis being the main side effects, occurring in a small percentage of the patients [34].

Several studies have proposed distinct cellular/molecular mechanisms to explain the chemopreventive effects of this group of compounds, particularly in other cancer types, such as the pancreatic and the prostate cancer, including the action on cell cycle and the induction of apoptosis and growth suppression [35–37]. Not excluding other eventually relevant contributions, such as the modulation of Ras and Rho proteins by statins-induced mevalonate depletion [38,39], our data suggest that Atorva use is able to promote cancer chemoprevention by anti-proliferative, anti-inflammatory and antioxidant actions. Serum TGF-β1, TNF-α and CRP levels were highly reduced when compared with the carcinogen (BBN) group. The anti-proliferative effect of atorvastatin was already demonstrated in two human transitional cell carcinoma cell lines (RT4 and KU-7) [28], as well as in a rat model of liver cancer [40]. Statins exert anti-inflammatory effects in numerous tissues via the activation of peroxisome proliferator-activated receptors (PPARs), which regulates important cellular functions, such as glucose and lipid catabolism, cellular differentiation, proliferation and survival [41–43], and has been viewed as promising therapeutic target for bladder cancer [44]. Furthermore, the effects of statins, namely of atorvastatin, seem to be synergistically increased by cycloxygenase (COX) 2 inhibitors [45], which was also effective in bladder cancer prevention in a previous study from our group, as well as in other studies [15,46]. This data reinforces the role played by inflammation in bladder carcinogenesis and the putative relevance of preventive strategies based on anti-inflammatory activity, such as demonstrated by atorvastatin in the present study.

CD31 is a 130 kDa integral membrane protein, also known as PECAM-1 (platelet endothelial cell adhesion molecule-1), a member of the immunoglobulin superfamily, which mediates cell-to-cell adhesion, and is used as a marker of vascularization/angiogenesis. New vessels allow tumors to grow and cells to escape into the circulation and lodge in other organs. CD31 in bladder carcinoma has been correlated with the tumor grade and stage [47]. In our study, however, although a trend to higher expression of CD31 in the BBN group was found, no significant differences were found between groups. The tumor suppressor p53 has a key role in cancer, namely by regulating cell cycle progression, apoptosis, or senescence in response to various stress signals. Inactivation of the p53 pathway accounts for one of the most common molecular defects in human cancers, including in urothelial cancer [48]. Our data is in agreement with an impairment of p53 expression in bladder cancer, viewed by increased immunostaining in the BBN-treated rats, both in pre-neoplastic (hypertrophic) and neoplastic (papillary tumors) regions. This effect was clearly prevented in the Atorva + BBN-treated animals. Ki67 is a nuclear protein which is expressed in cycling cells but not in quiescent cells, thus representing a measurement of the tumor growth fraction and has been used in bladder cancer as an index of proliferative activity [49]. In our study, the BBN animals showed increased ki67 immunostaining, both in hypertrophic and neoplastic (papillary tumors) areas, suggesting a role for proliferation in tumor growth. Atorvastatin treatment prevented the effect, reinforcing an anti-proliferative effect of this drug in the nitrosamine-induced bladder cancer.

The anti-inflammatory and anti-proliferative actions of atorvastatin were accompanied by a more beneficial redox status profile, demonstrated by the higher serum total antioxidant status and lower MDA/TAS ratio, most likely indicative of reduced reactive oxygen species (ROS) formation. Statins have been previously shown to have important anti-inflammatory, anti-proliferative and antioxidant properties [20–23]. Considering that these are key fuels for cancer development [50], and that antioxidant agents protect against bladder cell death [51], the inhibition of those pathways in very early stages of the carcinogenesis process seems to be crucial for the prevention of tumor growth.

## 4. Experimental Section

### 4.1. Animals and Groups

Male Wistar rats, aged 10 weeks (weighing *ca*. 250 g), purchased from Charles River Lab. Inc. (Barcelona, Spain), were maintained in an air conditioned room, subjected to 12 h dark/light cycles and given standard laboratory rat chow (SAFE A-04, Augy, France) and free access to tap water. Animal experiments were conducted according to the European Communities Council Directives on Animal Care and approved by our National and Institution’s Ethics Committee for animal Research. The animals were divided into four groups: (1) Control group (*n* = 8): vehicle only; (2) Atorvastatin (Atorva) group (*n* = 8): 3 mg/kg bw/day of Atorvastatin (Zarator®, Pfizer, Lisbon, Portugal); (3) Carcinogen (BBN) group (*n* = 20): 0.05% of *N*-butyl-*N*-(4-hydroxybutyl)nitrosamine (Tokyo Chemical Industry Co., Ltd, Tokyo, Japan); (4) Preventive Atorvastatin group (*n* = 8): Atorva (3 mg/kg bw/day) + BBN. Treatments were performed in a two step protocol: Phase 1: week 1 to week 8, animals from groups 3 and 4 received BBN 0.05% ad libitum in drinking water. Atorva was given to groups 2 and 4; Phase 2: week 9 to 20, for study tumor expression or chemoprevention and underlying mechanisms. BBN was given in drinking water and Atorva (prepared in vehicle) was given by an esophageal cannula. All animals completed the 20-week study protocol. Body weight was measured weekly and drink beverage was monitored during the experimental period.

### 4.2. Sample Collection and Preparation

Blood: Before sacrifice, rats were anesthetized by intraperitoneal injection of 2 mg/Kg of a 2:1 (*v*/*v*) 50 mg/mL Ketamine (Ketalar^®^, Parke-Davis, Pfizer Lab., Seixal, Portugal) solution in 2.5% chlorpromazine (Largatil^®^, Rhône-Poulenc Rorer, Vitória lab., Amadora, Portugal). Blood samples were immediately collected from the jugular vein using needles with no anticoagulant.

Tissues: Animals were sacrificed by cervical dislocation and the lungs, stomach, liver, kidneys and intestines were immediately removed, weighed and placed in ice-cold Krebs buffer or formaldehyde. Before removal, bladders were filled with a buffered formaldehyde solution and the urethra was tied, as pre-fixation for histological analysis.

### 4.3. Tumor Chemoprevention Analysis

#### 4.3.1. Quantitative Analysis (Number and Tumor Volume)

Each bladder pre-fixated in formaldehyde was carefully open; the lumen was inspected for grossly visible lesions and the number of tumors and tumor volume were reported in order to calculate the mean volume per rat and per tumor and the percentage of tumor per group.

#### 4.3.2. Qualitative Analysis (Bladder Histology)

The bladder was immersion-fixed in 4% buffered formaldehyde and processed for paraffin sectioning. Three slices from each bladder were embedded and 3 μm thick sections were stained with haematoxylin & eosin (H & E) and examined for histology.

### 4.4. Proliferation, Inflammation and Redox Status Markers

Serum levels of TGF-β1 and TNF-α were measured through an ultrasensitive Quantikine^®^ ELISA kits (R & D Systems, Minneapolis, USA). Serum CRP was measured by using an ELISA kit from Helica Biosystems, Inc. (Fullerton, CA, USA). The thiobarbituric acid reactive-species (TBARs) assay was used to evaluate serum lipid peroxidation, via MDA, and TAS in serum was measured through ferric reducing antioxidant potential (FRAP) assay, as previously described [52].

### 4.5. Bladder Cancer p53, ki67 and CD31 Immunohistochemistry

Immunohistochemical (IHC) staining of p53, ki67 and CD31 in bladder cancer tissue was performed in paraffin-embedded tissue, which was cut into 4 mm sections and mounted on polisinated slides, using standard staining procedures, as described previously [53–55]. For each case, a single representative slide was selected for staining and histologic evaluation. Briefly, slides were deparaffinised and hydrated with water. Antigen enhancement was performed by pre-treating with microwave heating in a citrate buffer, pH 6.00 (for three pulses of 5 min each at 250 W). The slides were washed three times, 2 min each, and then incubated with blocking serum for 10 min to block the nonspecific binding, and the excess of blocking serum was removed. Staining was performed using the primary monoclonal antibodies for p53, ki67 and CD31. An appropriate positive control was used in each staining run, and each slide was stained with a negative control obtained by omitting the primary antibody. Diaminobenzidine was used as chromogen. Standard procedures were used for visualisation and the intensity and/or percentage of positive staining in the dominant pattern within the tumor was graded on a semiquantitative scale (0–3), in which 0 is very low intensity and <25% of staining; 1 is mild and between 25 and 50%; 2 is moderate and between 50 and 75% and 3 is severe and >75% of staining. All slides were reviewed independently by 2 investigators blinded to the data.

### 4.6. Biochemical Assays (Safety Profile)

Serum creatinine, urea, AST, ALT and CK levels were assessed through automatic validated methods and equipments (Hitachi 717 analyser, Roche Diag. Inc., MA, USA).

### 4.7. Statistical Analysis

For statistical analysis we used the GraphPad Prism version 5.00 software from GraphPad Software (San Diego, California, USA). Results are means ± standard error of means (SEM). Comparisons between groups were performed using ANOVA with Bonferroni or Fisher post-hoc test. A *p* value less than 0.05 was considered statistically significant.

## 5. Conclusions

Our data shows the chemopreventive potential of an early atorvastatin treatment, due to its antioxidant, anti-proliferative and anti-inflammatory properties. This is particularly relevant for patients with bladder cancer risk factors as well as for those with previous episodes of bladder tumor, due to the high recurrence rates. In conclusion, apart from the known reduction of cardiovascular risk given by its anticholesterolaemic action, Atorvastatin might have further beneficial effects due to chemopreventive properties against of bladder cancer.

## Figures and Tables

**Table 1:** Macroscopic and microscopic evaluation of urothelial lesions.

Macroscopy (quantitative)	Control (*n* = 8)	Atorva (*n* = 8)	BBN (*n* = 20)		Atorva + BBN (*n* = 8)	
Tumor number						
% rats with tumor	0	0	65.0% (13 in 20)		12.5% (1 in 8)	
Number tumors/rat	0	0	1.2 ± 0.1 (16 in 13)		2.0 (2 in 1)	
Tumor volume (mm^3^):						
Mean/rat	0	0	138.5 ± 7.5 (in 13)		4.7 (in 1)	
Mean/tumor	0	0	112.5 ± 6.4 (in 16)		2.3 ± 0.2 (in 2)	

**Microscopy (qualitative)**	**Control**	**Atorva**	**BBN**		**Atorva + BBN**	

			**Tumors Group**	**Total Group**	**Tumors Group**	**Total Group**

Pre-neoplastic lesions						
Hyperplasia	0	0	100 (13/13)	100 (20/20)	100 (1/1)	38 (3/8)
High-grade dysplasia	0	0	100 (13/13)	75 (15/20)	100 (1/1)	13 (1/8)
Low-grade dysplasia	0	0	0 (0/13)	25 (5/20)	0 (0/1)	25 (2/8)
Neoplastic lesions						
Papillary tumor	0	0	100 (13/13)	65 (13/20)	100 (1/1)	13 (2/8)
Infiltrative tumor	0	0	15 (2/13)	10 (2/20)	0 (0/1)	0 (0/8)
Carcinoma *in situ*	0	0	31 (4/13)	20 (4/20)	0 (0/1)	0 (0/8)

**Table 2:** Safety profile: renal and liver function data and creatine kinase activity.

Parameters	Control (*n* = 8)	Atorva (*n* = 8)	BBN (*n* = 20)	Atorva + BBN (*n* = 8)
Renal function				
Creatinine (μmol/L)	50.39 ± 0.88	56.57 ± 0.88	54.81 ± 1.77	59.23 ± 3.54
Urea (mmol/L)	6.40 ± 0.31	5.99 ± 0.32	6.27 ± 0.16	6.85 ± 0.24
Liver function				
AST (IU/L)	51.57 ± 1.09	54.00 ± 2.31	76.78 ± 4.40 aaa	111.25 ± 5.72 bbb
ALT (IU/L)	30.86 ± 1.75	31.00 ± 1.00	36.17 ± 2.32	53.43 ± 6.10 bb
CK activity (U/L)	165.80 ± 14.60	697.67 ± 59.22 a	231.88 ± 8.22	1132.17 ± 206.39 bbb

Values are mean ± s.e.m.

a *p* < 0.05 and

aaa *p* < 0.001 *vs.* the Control group;

bb *p* < 0.01 and

bbb *p*< 0.001 *vs.* the BBN group.
